# Spiral volumetric optoacoustic tomography visualizes multi-scale dynamics in mice

**DOI:** 10.1038/lsa.2016.247

**Published:** 2017-04-07

**Authors:** X Luís Deán-Ben, Thomas F Fehm, Steven J Ford, Sven Gottschalk, Daniel Razansky

**Affiliations:** 1 Institute for Biological and Medical Imaging (IBMI), Helmholtz Center Munich, 85764 Neuherberg, Germany; 2 School of Medicine and School of Bioengineering, Technical University of Munich, 81675 Munich, Germany

**Keywords:** multi-scale dynamics, multi-spectral imaging, optoacoustic tomography, real-time imaging, whole-body imaging

## Abstract

Imaging dynamics at different temporal and spatial scales is essential for understanding the biological complexity of living organisms, disease state and progression. Optoacoustic imaging has been shown to offer exclusive applicability across multiple scales with excellent optical contrast and high resolution in deep-tissue observations. Yet, efficient visualization of multi-scale dynamics remained difficult with state-of-the-art systems due to inefficient trade-offs between image acquisition time and effective field of view. Herein, we introduce the spiral volumetric optoacoustic tomography technique that provides spectrally enriched high-resolution contrast across multiple spatiotemporal scales. *In vivo* experiments in mice demonstrate a wide range of dynamic imaging capabilities, from three-dimensional high-frame-rate visualization of moving organs and contrast agent kinetics in selected areas to whole-body longitudinal studies with unprecedented image quality. The newly introduced paradigm shift in imaging of multi-scale dynamics adds to the multifarious advantages provided by the optoacoustic technology for structural, functional and molecular imaging.

## Introduction

Progress in life sciences is directly linked to the ability to non-invasively track dynamic functional and molecular events in the unperturbed environment of an intact living organism^1^. Yet, *in vivo* imaging across multiple temporal scales is commonly associated with hard compromises between the achievable field of view (FOV), spatial resolution and overall image quality. For instance, magnetic resonance imaging (MRI) is capable of imaging whole mammal organisms with high spatial resolution but its temporal resolution is limited so that real-time imaging is only possible in single two-dimensional slices over confined FOVs^2^. At the other end of the electromagnetic spectrum, optical imaging modalities suffer from intense light scattering and poor spatial resolution when applied to whole vertebrate organisms^3, 4^. In general, a multi-modality approach has been traditionally employed to acquire information at multiple time scales by, for example, combining ultrasonography for fast dynamic imaging of specific areas with images of larger regions acquired by means of whole-body imaging modalities, such as MRI or computed tomography (CT)^5, 6^. However, the fundamentally different contrast mechanisms, sensitivity and other imaging metrics associated with different modalities often hamper efficient combination of the information obtained at several spatiotemporal scales^7^.

Optoacoustic imaging is experiencing a surge in pre-clinical research mainly due to its unique capacity to bridge the gap between the micro- and macro-scopic realms with the same type of contrast^8, 9^. As opposed to the severe resolution degradation with depth experienced by the optical imaging methods due to light scattering^10^, the spatial resolution of optoacoustics is mainly determined by ultrasonic propagation, so that high-resolution information deep from scattering tissues can be retrieved^11^. Moreover, in addition to providing excellent spectroscopic optical absorption contrast from endogenous tissue chromophores such as hemoglobin^12^ and melanin^13, 14^, optoacoustics can deliver highly specific information from targeted and activatable probes^15, 16^ as well as from genetic labels *in vivo*^17, 18, 19, 20^. To this end, various implementations of optoacoustic tomography systems have demonstrated both cross-sectional (2D) and volumetric (3D) imaging capabilities with temporal resolution in the tens of milliseconds range^21, 22, 23, 24, 25^. Thus, the ability to capture multi-spectral information from entire tissue volumes in real time has effectively rendered optoacoustics a five-dimensional imaging modality^26, 27^.

Yet, efficient visualization of multi-scale dynamics remained difficult with state-of-the-art systems due to inefficient trade-offs between image acquisition time and effective FOV. Although it was shown possible to render three-dimensional (3D) optoacoustic images from whole mice or entire organs with sub-millimeter spatial resolution^28, 29, 30^, full tomographic rotation was required for volumetric image acquisition in those systems precluding their use for real-time 3D visualization. In addition, as a large portion of the imaged mouse was concurrently illuminated and extensive signal averaging was necessary to overcome noise due to the relatively low light fluence levels. Alternatively, two-dimensional whole-body imaging systems^31^, in particular multi-spectral optoacoustic tomography small animal scanners^21^, were reported to deliver entire mouse cross-sections in real time while the images were also combined into 3D whole-body views^32^. Yet, the inherently two-dimensional tomographic geometry of those systems hindered real-time 3D image acquisition, whereas the image quality further suffered from highly anisotropic resolution due to the use of focused detectors.

Herein, we developed a spiral volumetric optoacoustic tomography (SVOT) technique that offers the unique capability to efficiently bridge the visualization of dynamic processes at multiple scales. The temporal resolution of the system inversely scales with the size of the scanned region, whereas real-time 3D imaging remains possible for an area effectively captured by the volumetric imaging probe. By means of SVOT, we demonstrate a range of multi-scale dynamic imaging capabilities, from 3D millisecond-scale visualization at the whole-organ level, via tracking contrast agent kinetics in selected areas, all the way to whole-body longitudinal studies with unprecedented image quality.

## Materials and methods

### The SVOT imaging concept

**Figure 1:**
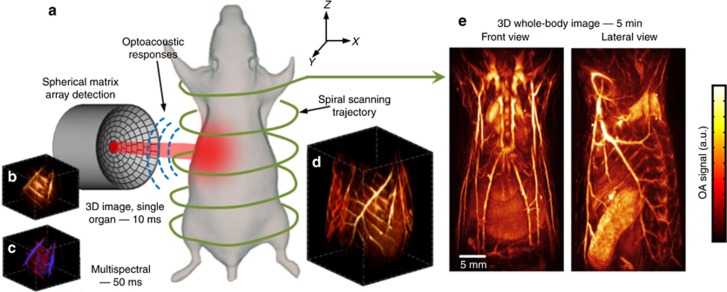
The spiral volumetric optoacoustic tomography (SVOT) approach. (**a**) Whole-body tomographic data acquisition is performed along a spiral (helical) scanning trajectory by means of a spherical matrix ultrasound detection array, further capable of real-time three-dimensional (3D) imaging. (**b**) Representative single 3D image acquired from the mouse’s abdomen. The system can render single (~1 cm^3^) volumes at a frame rate of 100 Hz, only limited by the pulse repetition rate of the excitation laser. (**c**) Multi-spectral (or spectrally unmixed) 3D images can be generated for each position of the matrix array after acquiring volumetric image data at multiple excitation wavelengths of the laser. By fast sweeping of the laser wavelength, it only takes 50 ms to generate a volumetric multi-spectral dataset at five wavelengths. (**d**) Volumetric optoacoustic image rendered from a larger area by performing a partial scan for 5 s. (**e**) It takes ~5 min to acquire whole-body image data by combining all images acquired along the entire spiral trajectory.

The tomographic imaging concept of SVOT is schematically depicted in Figure 1a, whereas a detailed layout of the experimental system is further provided in Supplementary Fig. 1a. Tissue excitation is done with an unfocused beam of short-pulsed laser light in the near-infrared spectrum, which diffuses into deep-tissue layers generating ultrasound (optoacoustic) waves via thermal expansion^11^. The optical parametric oscillator-based laser, used as an optoacoustic excitation source, provides <10 ns duration pulses with energies approaching 30 mJ and a pulse repetition frequency of up to 100 Hz. The light beam was guided through a custom-made fiber bundle (CeramOptec GmbH, Bonn, Germany) inserted through an opening in the center of the active surface of the array. The light coupling efficiency was ~60%, whereas a Gaussian illumination profile with a size of 10 mm at the full width at half maximum (FWHM) was created at the tissue surface by the bundle. The laser fluence levels at the mouse surface were below 20 mJ cm^−2^. The excited optoacoustic responses were collected at multiple locations around the imaged volume with a sensitive 256-element spherical matrix detection array of 40 mm radius, which allowed for on-the-fly image reconstruction and rendering of a volumetric (3D) image of a specific region after each laser pulse. The individual elements of the transducer array have an approximate area of 9 mm^2^, central detection frequency of 4 MHz and a −6 dB bandwidth of 100%. The effective size of the FOV captured for every laser shot was previously determined to be ~1 cm^3^ according to the FWHM of the combined sensitivity field of the entire matrix array. An isotropic resolution of ~200 μm was measured around the geometrical center of the spherical array, degrading to ~600 μm at the periphery of the FOV^33^. The optoacoustic signals were digitized at 40 mega samples per second with a custom-made data acquisition system (Falkenstein Mikrosysteme GmbH, Taufkirchen, Germany) triggered with the Q-switch output of the laser. In this way, the maximum imaging frame rate of 100 volumes per second (determined by the pulse repetition frequency of the laser) is achieved for the ~1 cm^3^ FOV—size of a typical mouse organ (Figure 1b). In addition, multi-spectral information from the imaged volume (Figure 1c) is readily collected by fast sweeping of the laser wavelength, which can be tuned, on a per-pulse basis, to any desirable value within the near-infrared spectral window (680–950 nm).

Imaging at multiple spatiotemporal scales is realized by fast motion of the spherical detection array and acquisition of multiple volumetric data sets along a spiral (helical) trajectory, where a larger FOV comes at expense of the temporal resolution (Figure 1d and 1e). This was realized by scanning the spherical array using a rotation stage (Model: RCP2-RTCL-I-28P-30-360, IAI Inc., Shizuoka Prefecture, Japan) and a translation stage (Model: RCP2-RGD6C-I-56P-4, IAI Inc.), whereas the relative *x*–*y* position of the focus of the spherical array could be further varied with respect to the rotational axis by means of two additional manual translation stages. The radius and pitch of the helical scanning geometry were set to 45 and 2 mm, respectively. Both the data acquisition and the motorized stages were controlled with a personal computer using a custom MATLAB interface (MathWorks Inc., Natick, USA).

### Image reconstruction and spectral unmixing

Image reconstruction of the individual volumetric frames was performed with a graphics processing unit implementation of a back-projection reconstruction formula^34^. The time-resolved signals were deconvolved with the frequency response of the ultrasound sensors and band-pass filtered with cutoff frequencies 0.1 and 7 MHz. Image rendering from large regions was performed by stitching the volumetric images acquired at each position of the array. The stitching method simply consists in adding up volumes acquired from the individual laser shots after proper rotation and translation. Note that data from the individual laser shots contains no information from unilluminated areas, making the volume stitching method practically equivalent to simultaneously reconstructing whole-body images from all the projections. For the stitching, the position and orientation of the spherical array with respect to the rotation axis (parameters *r*, *l* and *ϕ* in Supplementary Fig. 1b) were accurately calibrated before each experiment by full-view tomographic scanning of an agar phantom containing a 100 μm diameter absorbing microsphere. Full characterization of the resolution of the system was further performed by translating the phantom within a cylindrical region of 28 mm diameter and 15 cm height, rendering spatial resolution performance better than 250 μm (lateral *x*–*y* plane) and 500 μm (vertical *z* axis) within the entire region. The slightly better resolution performance in the lateral dimension is generally expected due to the full tomographic coverage in the *x*–*y* plane. In the process of whole-body image rendering, a clustering approach was further applied to remove the influence of inter-frame motion artifacts due to breathing. For this, a correlation matrix between consecutive frames was calculated and a k-means sorting algorithm was applied to retain only those frames not affected by respiratory motion. Unmixing the distribution of specific chromophores was performed by least-square spectral fitting of the individual voxels reconstructed at multiple wavelengths using the molar extinction coefficients of the chromophore of interest and oxygenated and deoxygenated hemoglobin^35^.

### Animal handling and contrast agent injection

All procedures involving animal care and experimentation were conducted in full compliance with the institutional guidelines of the Institute for Biological and Medical Imaging and with approval from the Government District of Upper Bavaria. The imaged mouse was placed in a specifically designed holder (Supplementary Fig. 1c) attached to the bottom of a water tank. The mouse remained in a stationary position through the tomographic data acquisition with its fore and hind paws attached to the holder and a mouth clamp used for coupling to the gas anesthesia mask. The water in the tank was kept at a constant temperature of 34 °C during the scan using a feedback-controlled heating stick. *In vivo* experiments were performed under isoflurane anesthesia (2–3% v/v) in 100% O_2_. The contrast agents were diluted in phosphate-buffered saline (PBS) and injected intravenously (i.v.) through a tail-vein catheter. Specifically, 100 nmol of indocyanine green (ICG) diluted in 100 μl of PBS were injected for imaging heart dynamics, 100 nmol of ICG diluted in 100 μl of PBS were injected for imaging tumor perfusion and 10 nmol of AlexaFluor 750 (AF750) diluted in 100 μl of PBS were injected for imaging renal clearance.

### Estimation of the clearance constants

The decay and increase rates associated with the concentration of injected contrast agents were estimated by assuming a mono-compartmental pharmacokinetic model (first-order kinetics) for the analyzed regions^36^. With this approach, the elimination rate of the contrast agent from the vasculature was assumed to be proportional to its concentration in defined vascular structures. The temporal profile of the concentration of the probe of interest was then further considered to be proportional to the difference between the time-dependent optoacoustic signal and its background level before injection. Thereby, the temporal dependence of the optoacoustic signal intensity at a given blood vessel OA_v_(*t*) can be expressed as


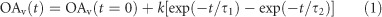


The term exp(−*t/τ*_2_) in Equation (1) was included to account for the time required for the contrast agent to uniformly dilute in blood, that is, it represents a one-compartmental model for the elimination of the contrast agent bolus from the injection spot into the blood circulation. The decay rate of the probe concentration in a given voxel is then associated to the time constant *τ*_1_. The decay rates of AF750 in the renal artery and the kidney cortex as well as the decay rate of ICG in the tumor area were then estimated by fitting the time profiles of the optoacoustic signals to Equation (1). Different time constants in these regions correspond to different elimination mechanisms of the contrast agent. For example, AF750 is eliminated from the renal artery to the kidney cortex and from the kidney cortex capillaries to the renal pelvis. On the other hand, the decay rate in the tumor region is associated to a combination of ICG leakage into the extracellular matrix due to enhanced permeability and retention effect^37^ as well as its clearance from the blood circulation into other organs.

The accumulation rate of ICG in the liver was also estimated with the same model. Assuming that ICG is eliminated from the blood according to Equation (1), the optoacoustic signal intensity in the liver OA_l_(*t*) due to accumulation of the probe was modeled by integrating this expression considering 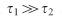 as


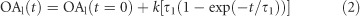


The time constant *τ*_1_ obtained by fitting the time profile of the optoacoustic signal to Equation (2) was then taken as the rate of ICG increase in the liver.

### Blood flow velocity estimation

The blood flow velocity can be estimated from the delays in the ICG bolus appearance, as measured in the corresponding time-dependent optoacoustic signals. This approach is applicable if the flow velocity is sufficiently slow so that a delay can be measured from voxels rendered in real time at the specific position of the transducer array. To estimate this delay, an 80-point moving average filter was first applied to the optoacoustic signals, which were subsequently normalized to their maximum and minimum values. The normalized signals were then fitted to a fifth-order polynomial for the relevant time intervals. The delays were estimated from the instants at which the fitted polynomials equal 0.5. The delay Δ*t*_ca_ between the ICG bolus appearance at the thoracic artery and the coronary artery was further used to estimate the blood flow velocity in the coronary artery. Considering that the selected point in the coronary artery is located at a distance *d*~3 mm from the aortic branch, the flow velocity *v* was estimated via *v*=*d*/Δ*t*_ca_.

### Tumor model

An 8-week-old female athymic nude-Fox1^nu^ mouse (Harlan Laboratories LTD, Itingen, Switzerland) was used for tumor inoculation. An orthotopic tumor was grown upon subcutaneous injection of 0.5 × 10^6^ 4T1 murine breast cancer cells into the thoracic mammary fat pad. Imaging experiments were performed at days 6, 8 and 11 post inoculation. The diameter of the tumor was ~8 mm at day 11. The athymic nude-Fox1^nu^ mutant nude mice strain represents a suitable allograft host, as defects in the immune system enable an effective and reproducible tumor growth. Furthermore, the lack of highly absorbing hair and melanin skin pigmentation makes the strain ideal for reducing artefacts in the optoacoustic images.

## Results and discussion

The suggested tomographic scanning geometry of SVOT has demonstrated a unique capacity for whole-body imaging of the optical absorption distribution with unprecedented image quality (Figure 1e; Supplementary Fig. 2; Supplementary Movies 1 and 2), while further retaining real-time imaging capacity at the single-organ level (Figure 1b) as well as high temporal resolution in 3D observations from limited areas (Figure 1d). Naturally, the maximum intensity projection views can mitigate the presence of noise and other artefacts in the images and further make the superficial areas more prominent due to light attenuation in deep tissues. We therefore included individual cross-sections (Supplementary Fig. 2g; Supplementary Movie 3) to showcase the actual depth dependence of the reconstructed signals. Although part of cross-sections contain visible contrast across all depths, presence of highly vascularized organs (such as the liver) in other slices limits visibility to only superficial layers due to the strong light attenuation.

In the present study, all reconstructions were performed assuming homogeneous speed of sound distribution and no acoustic attenuation or scattering. The image quality can be potentially enhanced by accounting for speed of sound variations and acoustic attenuation, although we have previously shown that these effects have a limited impact on the images acquired from soft tissues for the 4 MHz frequency range of the transducer array employed here^38, 39^. Acoustic heterogeneities may however become dominant at higher ultrasound frequencies, and more advanced reconstruction algorithms accounting for these effects^40, 41, 42, 43^ may turn beneficial if a high-resolution imaging system is developed. Note that strong distortions are produced for all the generated ultrasound frequencies in the lung region due to the enormous acoustic mismatches at the air cavities^44^.

**Figure 2:**
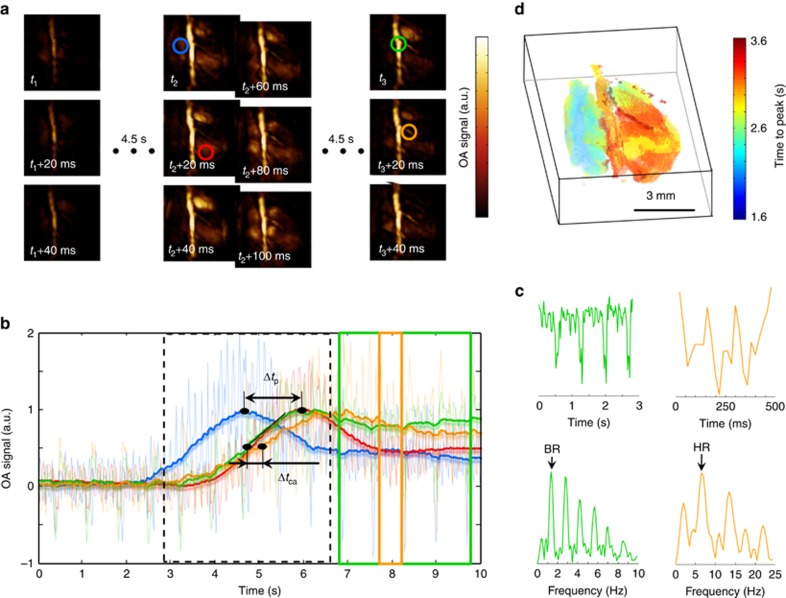
High-frame-rate volumetric imaging at the whole-organ level. (**a**) Maximum intensity projection (MIP) of the three-dimensional (3D) optoacoustic images acquired from a living murine heart after tail-vein injection (at *t*_1_=0 s) of 100 nmol of indocyanine green (ICG). (**b**) Optoacoustic signal traces (dimmed lines) along with their respective low-pass-filtered equivalents (bold lines) for the respective regions indicated in **a**. The pulmonary transit time Δ*t*_p_ and the delay Δ*t*_ca_ between the ICG appearance at a coronary artery versus the thoracic artery are indicated. (**c**) Zoom into the signal traces marked with equivalent color in **b** along with their respective Fourier transforms—the two distinctive peaks correspond to the breathing rate (BR) and heart rate (HR). (**d**) 3D color mapping of the time-to-peak values corresponding to the ICG bolus appearance.

We then exploited the high volumetric frame rate of the system for *in vivo* imaging of murine cardiac dynamics. Figure 2a shows a fast temporal sequence of volumetric images acquired from the mouse heart at 800 nm after tail-vein injection of ICG. The heart motion registered to the whole-body anatomical image can be best visualized in the video available in the on-line version of the journal (Supplementary Movie 4). The optoacoustic signal traces from individual voxels along with their low-pass-filtered equivalents (bold lines) are plotted in Figure 2b. Fourier analysis of the signals at different temporal scales readily reveals the cardiac (7.1 Hz) and respiratory (1.4 Hz) rates to be in the normal physiological range. High-frame-rate volumetric heart imaging on a beat-by-beat basis further enables a clear separation of heart perfusion dynamics from the faster cardiac and respiratory motion. For this, the low-pass-filtered signal profiles were used to estimate the time-to-peak values for each image voxel, as shown in Figure 2d. This enables the segmentation of the heart chambers based on the timing of the ICG bolus appearance. By considering the difference in time-to-peak values for the voxels located in the right versus left ventricles, the estimated value of the pulmonary transit time Δ*t*_p_=1.24 s again belongs to the normal physiological range, but may potentially serve as a useful indicator of ventricular dysfunction. Note that this type of 3D analysis of fast contrast agent perfusion on a 10 ms temporal scale is not possible with other cardiac imaging modalities, such as MRI or CT, which commonly rely on gated data acquisition or other retrospective gating approaches for high-frame-rate visualization of the beating heart in 3D. To further characterize the blood flow, we measured a delay of Δ*t*_ca_=300 ms between the ICG bolus appearance at the thoracic artery and a coronary artery, which translates into 10 mm s^−1^ flow velocity in the coronary artery (see the Materials and methods section).

**Figure 3:**
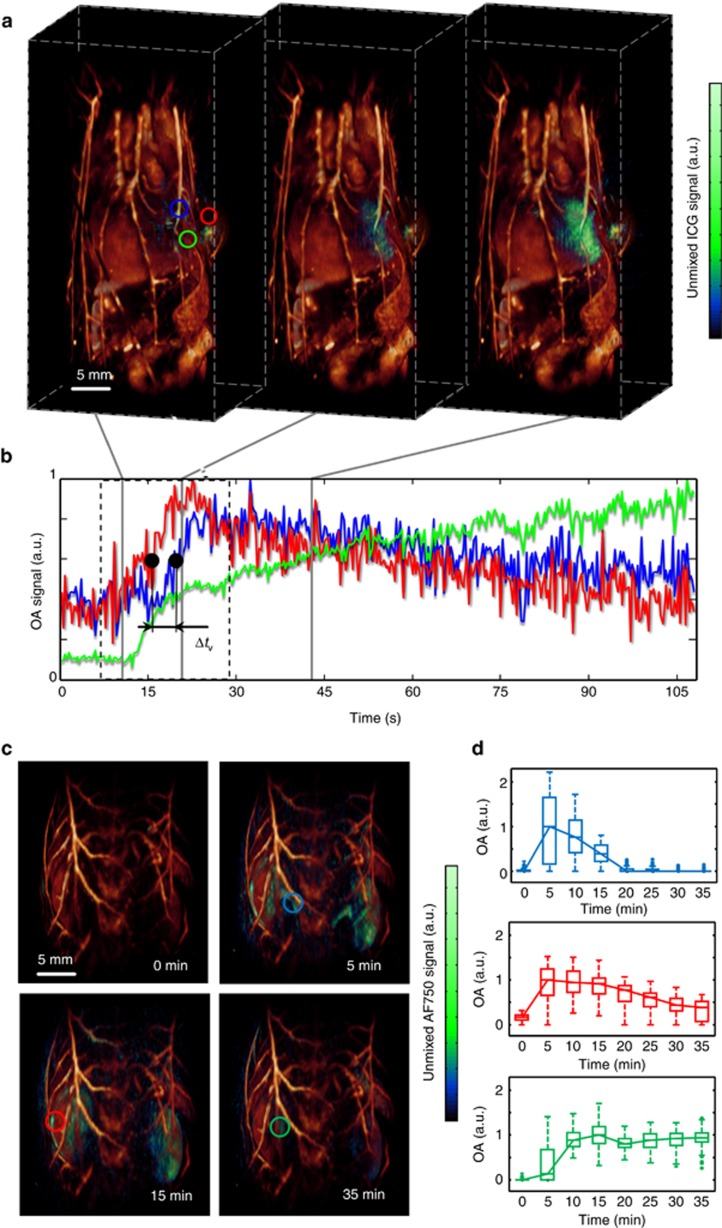
Contrast agent kinetics in larger areas. (**a**) Spectrally unmixed images (green) for three representative instants following tail-vein injection of 100 nmol of the agent at 0 s. The kinetic images were superimposed onto the whole-body optoacoustic anatomical reference (acquired at 800 nm wavelength). (**b**) Optoacoustic signal traces from the regions indicated by circles in **a**. Note the indicated delay Δ*t*_v_ between the indocyanine green (ICG) appearance in a lateral vein versus the tumor area. (**c**) Spectrally unmixed images (green) superimposed onto an anatomical reference image of the entire kidney region. Tail-vein injection of 10 nmol was done at *t*=0 s. (**d**) Box plots indicate distribution of the optoacoustic signal values in the corresponding volumes of interest labeled in **c**.

The sensitivity and specificity in detecting spectrally distinct contrast agents can be further enhanced when employing a multi-spectral data acquisition approach^26^, in which case the attained frame rate is accordingly reduced by the number of wavelengths employed. Figure 3a displays a time-lapse sequence of spectrally unmixed kinetics of ICG after injecting 100 nmol of the agent into the tail vein of a tumor-bearing mouse. Unmixing was performed with optoacoustic images at 730, 760, 800, 850, and 900 nm. A more comprehensive visualization of the agent kinetics is available in the on-line video (Supplementary Movie 5). As SVOT has the powerful ability to acquire a high-resolution whole-body image of the same mouse, the latter serves here as a valuable anatomical reference for interpretation of the fast 3D kinetic data. We further analyzed the differential kinetic profiles in the tumor area (Figure 3b). Noticeable is the delay of Δ*t*_v_=1.26 s between the contrast agent appearance in a major lateral vein versus the tumor area, most likely ascribed to vein obstruction caused by the tumor growth. For functional assessments of the throughput capacity of the tumor neovasculature, we further estimated contrast agent dynamics in the tumor area starting at *t*=30 s, yielding an average decay rate of 0.55 min^−1^ (see the Materials and methods section). The corresponding signal increase in a volume of interest located in the liver area amounted to 0.31 min^−1^ (see Materials and methods section), serving as an additional reference for the liver clearance rates and hepatic function^45^. We also tracked the longitudinal tumor development by acquisition of whole-body images from the same mouse over 11 days post inoculation (Supplementary Figure 3). This helped discerning anatomical transformations at the different stages of tumor progression, including blockage of the lateral vein, which has arguably caused the observed ICG kinetic changes.

The ability of SVOT to observe slower pharmacokinetics or longitudinal dynamics at the whole-organ system level is further showcased in Figure 3c and 3d and Supplementary Fig. 4. Here the unmixing for the AF750 component was performed with images acquired at 710, 730, 745, 765, 800, 850 and 900 nm. Renal clearance of small-molecule agents occurs on a timescale of minutes, which makes it possible for SVOT to observe metabolic profiles in the kidneys by measuring the unmixed bio-distribution of AF750 in a larger mouse abdomen region. Analysis of the signal profiles reveals that, although the amount of contrast agent in the renal artery area rapidly increases to a maximum value within 5 min post injection, then rapidly decaying at a rate of 0.094 min^−1^ (see the Materials and methods section), the corresponding clearance in the kidney cortex area was much slower (0.026 min^−1^ (see the Materials and methods section)). The third analyzed region corresponds to the renal pelvis, where AF750 is eliminated towards the ureter. In this area, the signal only starts increasing after 5 min post injection before reaching a plateau. The observed behavior is consistent with the normal metabolic function of the renal system, which includes infiltration of small molecules through the renal artery, filtration in the cortex and subsequent excretion towards the ureter.

It is important to emphasize that the results of the ICG and AF750 perfusion experiments are only suitable for qualitative assessment of the agent distribution. This is because wavelength-dependent attenuation of light causes alterations in the spectral profile of optoacoustic responses at deep locations^46^, which may lead to quantification errors in estimating the bio-distribution of specific absorbing agents and to a reduced sensitivity in their detection. Properly correcting for this effect implies solving non-linear inversion problems based on accurate knowledge of the highly heterogeneous optical properties of tissues^47^, whose 3D mapping in living organisms is challenging^48^. Although the standard linear unmixing approach employed in this work may be suitable for estimating temporal profiles at the relatively shallow locations analyzed in Figure 3 where light attenuation is not significant, other algorithms based on blind unmixing or statistical methods can be employed to resolve smaller amounts of contrast agents at deeper locations^46, 49^. Yet, presence of an exogenous contrast agent may generate additional alterations in the optical properties hence cause significant temporal variations in the light fluence distribution. Clearly, these temporal changes are also strongly dependent on the generally unknown temporal and 3D spatial bio-distribution profiles of the particular agent.

## Conclusions

By introducing the new SVOT technique, we demonstrated high-resolution imaging performance in living mice across multiple spatiotemporal scales using the same excellent contrast provided by optoacoustics. In contrast, the commonly adopted imaging paradigms rely on different systems to provide information at different spatiotemporal scales or otherwise use a complementary modality with a different type of contrast to support the multi-scale imaging capabilities. For instance, in cardiac imaging applications, the superior temporal resolution available on many ultrasound scanners can at least partially offset the advantage in spatial resolution and whole-body imaging capabilities enjoyed by high-field MRI scanners^50^. Yet, those combinations are often associated with inefficient trade-offs in terms of image acquisition time, effective FOV and/or contrast rendered with each particular system^7^. Our experiments in mice have attained a wide range of multi-scale imaging capabilities for the SVOT method, from beat-to-beat visualization of a beating heart at 100 Hz volumetric frame rate to slower contrast agent kinetics in selected areas at the entire renal system level to whole-body longitudinal studies of tumor growth with unprecedented image quality.

In conclusion, the ability to deliver high-resolution images at the whole-body scale while preserving ultrafast 3D imaging capability in smaller regions with the same type of contrast makes the SVOT technique unique among the existing pre-clinical imaging modalities. In addition, contrary to non-optical imaging methods, such as MRI, CT or ultrasonography, SVOT has the powerful contrast advantages of optical interrogation methods, including the ability to visualize rich functional and molecular information on blood oxygenation as well as targeted delivery of contrast agents and genetic labels^51^. Nonetheless, as opposed to optical microscopy, SVOT does not suffer from spatial resolution degradation due to light scattering in deep tissues, thus can also provide high-quality images with optical contrast at the whole-body level.

## Author contributions

XLDB and DR conceived the study. XLDB and TFF designed the optoacoustic imaging system. XLDB, TFF, SJF and SG carried out the experiments. TFF and XLDB implemented image reconstruction and processing algorithms and analyzed the data. DR supervised the study and data analysis. All authors discussed the results and contributed to writing the manuscript.

## References

[bib1] Weissleder R, Nahrendorf M. Advancing biomedical imaging. Proc Natl Acad Sci USA 2015; 112: 14424–14428. 26598657 10.1073/pnas.1508524112 PMC4664297

[bib2] Yu X, Qian CQ, Chen DY, Dodd SJ, Koretsky AP. Deciphering laminar-specific neural inputs with line-scanning fMRI. Nat Methods 2014; 11: 55–58. 24240320 10.1038/nmeth.2730 PMC4276040

[bib3] Ale A, Ermolayev V, Herzog E, Cohrs C, de Angelis MH et al. FMT-XCT: *in vivo* animal studies with hybrid fluorescence molecular tomography-X-ray computed tomography. Nat Methods 2012; 9: 615–620. 22561987 10.1038/nmeth.2014

[bib4] Eggebrecht AT, Ferradal SL, Robichaux-Viehoever A, Hassanpour MS, Dehghani H et al. Mapping distributed brain function and networks with diffuse optical tomography. Nat Photonics 2014; 8: 448–454. 25083161 10.1038/nphoton.2014.107 PMC4114252

[bib5] Wein WG, Brunke S, Khamene A, Callstrom MR, Navab N. Automatic CT-ultrasound registration for diagnostic imaging and image-guided intervention. Med Image Anal 2008; 12: 577–585. 18650121 10.1016/j.media.2008.06.006

[bib6] Hu YP, Ahmed HU, Taylor Z, Allen C, Emberton M et al. MR to ultrasound registration for image-guided prostate interventions. Med Image Anal 2012; 16: 687–703. 21216180 10.1016/j.media.2010.11.003

[bib7] Cherry SR. Multimodality *in vivo* imaging systems: Twice the power or double the trouble? Ann Rev BiomedEng 2006; 8: 35–62. 10.1146/annurev.bioeng.8.061505.095728 16834551

[bib8] Wang LV, Hu S. Photoacoustic tomography: *In vivo* imaging from organelles to organs. Science 2012; 335: 1458–1462. 22442475 10.1126/science.1216210 PMC3322413

[bib9] Yao JJ, Kaberniuk AA, Li L, Shcherbakova DM, Zhang RY et al. Multiscale photoacoustic tomography using reversibly switchable bacterial phytochrome as a near-infrared photochromic probe. Nat Methods 2016; 13: 67–73. 26550774 10.1038/nmeth.3656 PMC4697872

[bib10] Ntziachristos V. Going deeper than microscopy: the optical imaging frontier in biology. Nat Methods 2010; 7: 603–614. 20676081 10.1038/nmeth.1483

[bib11] Beard P. Biomedical photoacoustic imaging. Interface Focus 2011; 1: 602–631. 22866233 10.1098/rsfs.2011.0028 PMC3262268

[bib12] Yao JJ, Wang LD, Yang JM, Maslov KI, Wong TTW et al. High-speed label-free functional photoacoustic microscopy of mouse brain in action. Nat Methods 2015; 12: 407–410. 25822799 10.1038/nmeth.3336 PMC4428901

[bib13] Kirscher L, Deán-Ben XL, Scadeng M, Zaremba A, Zhang Q et al. Doxycycline inducible melanogenic vaccinia virus as theranostic anti-cancer agent. Theranostics 2015; 5: 1045–1057. 26199644 10.7150/thno.12533 PMC4508495

[bib14] Jathoul AP, Laufer J, Ogunlade O, Treeby B, Cox B et al. Deep *in vivo* photoacoustic imaging of mammalian tissues using a tyrosinase-based genetic reporter. Nat Photonics 2015; 9: 239–246.

[bib15] Razansky D, Harlaar NJ, Hillebrands JL, Taruttis A, Herzog E et al. Multispectral optoacoustic tomography of matrix metalloproteinase activity in vulnerable human carotid plaques. Mol Imaging Biol 2012; 14: 277–285. 21720908 10.1007/s11307-011-0502-6 PMC3346936

[bib16] Li JJ, Cheng FF, Huang HP, Li LL, Zhu JJ. Nanomaterial-based activatable imaging probes: from design to biological applications. Chem Soc Rev 2015; 44: 7855–7880. 26214317 10.1039/c4cs00476k

[bib17] Deliolanis NC, Ale A, Morscher S, Burton NC, Schaefer K et al. Deep-tissue reporter-gene imaging with fluorescence and optoacoustic tomography: a performance overview. Mol Imaging Biol 2014; 16: 652–660. 24609633 10.1007/s11307-014-0728-1

[bib18] Krumholz A, Shcherbakova DM, Xia J, Wang LV, Verkhusha VV. Multicontrast photoacoustic *in vivo* imaging using near-infrared fluorescent proteins. Sci Rep 2014; 4: 3939. 24487319 10.1038/srep03939 PMC3909896

[bib19] Gottschalk S, Estrada H, Degtyaruk O, Rebling J, Klymenko O et al. Short and long-term phototoxicity in cells expressing genetic reporters under nanosecond laser exposure. Biomaterials 2015; 69: 38–44. 26280948 10.1016/j.biomaterials.2015.07.051

[bib20] Jiang Y, Sigmund F, Reber J, Deán-Ben XL, Glasl S et al. Violacein as a genetically-controlled, enzymatically amplified and photobleaching-resistant chromophore for optoacoustic bacterial imaging. Sci Rep 2015; 5: 11048. 26091543 10.1038/srep11048 PMC4473533

[bib21] Razansky D, Buehler A, Ntziachristos V. Volumetric real-time multispectral optoacoustic tomography of biomarkers. Nat Protoc 2011; 6: 1121–1129. 21738125 10.1038/nprot.2011.351

[bib22] Nasiriavanaki M, Xia J, Wan HL, Bauer AQ, Culver JP et al. High-resolution photoacoustic tomography of resting-state functional connectivity in the mouse brain. Proc Natl Acad Sci USA 2014; 111: 21–26. 24367107 10.1073/pnas.1311868111 PMC3890828

[bib23] Xiang LZ, Wang B, Ji LJ, Jiang HB. 4-D photoacoustic tomography. Sci Rep 2013; 3: 1113. 23346370 10.1038/srep01113 PMC3552346

[bib24] Taruttis A, Ntziachristos V. Advances in real-time multispectral optoacoustic imaging and its applications. Nat Photonics 2015; 9: 219–227.

[bib25] Deán-Ben XL, Ford SJ, Razansky D. High-frame rate four dimensional optoacoustic tomography enables visualization of cardiovascular dynamics and mouse heart perfusion. Sci Rep 2015; 5: 10133. 26130401 10.1038/srep10133 PMC4486932

[bib26] Deán-Ben XL, Razansky D. Adding fifth dimension to optoacoustic imaging: volumetric time-resolved spectrally enriched tomography. Light Sci Appl 2014; 3: e137.

[bib27] Deán-Ben XL, Bay E, Razansky D. Functional optoacoustic imaging of moving objects using microsecond-delay acquisition of multispectral three-dimensional tomographic data. Sci Rep 2014; 4: 5878. 25073504 10.1038/srep05878 PMC4115207

[bib28] Brecht HP, Su R, Fronheiser M, Ermilov SA, Conjusteau A et al. Whole-body three-dimensional optoacoustic tomography system for small animals. J Biomed Opt 2009; 14: 064007. 20059245 10.1117/1.3259361 PMC2794413

[bib29] Kruger RA, Lam RB, Reinecke DR, Del Rio SP, Doyle RP. Photoacoustic angiography of the breast. Med Phys 2010; 37: 6096–6100. 21158321 10.1118/1.3497677 PMC2994935

[bib30] Gateau J, Caballero MAA, Dima A, Ntziachristos V. Three-dimensional optoacoustic tomography using a conventional ultrasound linear detector array: whole-body tomographic system for small animals. Med Phys 2013; 40: 013302. 23298121 10.1118/1.4770292

[bib31] Xia J, Chatni MR, Maslov K, Guo ZJ, Wang K et al. Whole-body ring-shaped confocal photoacoustic computed tomography of small animals *in vivo*. J Biomed Opt 2012; 17: 050506. 22612121 10.1117/1.JBO.17.5.050506 PMC3382342

[bib32] Buehler A, Deán-Ben XL, Razansky D, Ntziachristos V. Volumetric optoacoustic imaging with multi-bandwidth deconvolution. IEEE Trans Med Imaging 2014; 33: 814–821. 24058023 10.1109/TMI.2013.2282173

[bib33] Deán-Ben XL, Razansky D. Portable spherical array probe for volumetric real-time optoacoustic imaging at centimeter-scale depths. Opt Express 2013; 21: 28062–28071. 24514320 10.1364/OE.21.028062

[bib34] Deán-Ben XL, Ozbek A, Razansky D. Volumetric real-time tracking of peripheral human vasculature with GPU-accelerated three-dimensional optoacoustic tomography. IEEE Trans Med Imaging 2013; 32: 2050–2055. 23846468 10.1109/TMI.2013.2272079

[bib35] Deán-Ben XL, Razansky D. Functional optoacoustic human angiography with handheld video rate three dimensional scanner. Photoacoustics 2013; 1: 68–73. 25302151 10.1016/j.pacs.2013.10.002 PMC4134902

[bib36] Gerlowski LE, Jain RK. Physiologically based pharmacokinetic modeling: principles and applications. J Pharm Sci 1983; 72: 1103–1127. 6358460 10.1002/jps.2600721003

[bib37] Bertrand N, Wu J, Xu XY, Kamaly N, Farokhzad OC. Cancer nanotechnology: the impact of passive and active targeting in the era of modern cancer biology. Adv Drug Deliv Rev 2014; 66: 2–25. 24270007 10.1016/j.addr.2013.11.009 PMC4219254

[bib38] Deán-Ben XL, Razansky D, Ntziachristos V. The effects of acoustic attenuation in optoacoustic signals. Phys Med Biol 2011; 56: 6129–6148. 21873768 10.1088/0031-9155/56/18/021

[bib39] Deán-Ben XL, Ntziachristos V, Razansky D. Effects of small variations of speed of sound in optoacoustic tomographic imaging. MedPhys 2014; 41: 073301. 10.1118/1.4875691 24989414

[bib40] Treeby BE, Cox BT. k-Wave: MATLAB toolbox for the simulation and reconstruction of photoacoustic wave fields. J Biomed Opt 2010; 15: 021314. 20459236 10.1117/1.3360308

[bib41] Huang C, Nie LM, Schoonover RW, Wang LV, Anastasio MA. Photoacoustic computed tomography correcting for heterogeneity and attenuation. J Biomed Opt 2012; 17: 061211. 22734741 10.1117/1.JBO.17.6.061211 PMC3380947

[bib42] Jose J, Willemink RGH, Steenbergen W, Slump CH, van Leeuwen TG et al. Speed-of-sound compensated photoacoustic tomography for accurate imaging. Med Phys 2012; 39: 7262–7271. 23231277 10.1118/1.4764911

[bib43] Paltauf G, Wurzinger G, Nuster R. Speed-of-sound correction for photoacoustic and laser-ultrasound imaging with an integrating cylindrical detector. Proceedings of SPIE 9539, Opto-Acoustic Methods and Applications in Biophotonics II, 953917. Optical Society of America: Munich, Germany, 2015.

[bib44] Deán-Ben XL, Ma R, Rosenthal A, Ntziachristos V, Razansky D. Weighted model-based optoacoustic reconstruction in acoustic scattering media. Phys Med Biol 2013; 58: 5555–5566. 23892587 10.1088/0031-9155/58/16/5555

[bib45] Sakka SG. Assessing liver function. Curr Opin Crit Care 2007; 13: 207–214. 17327744 10.1097/MCC.0b013e328012b268

[bib46] Tzoumas S, Deliolanis NC, Morscher S, Ntziachristos V. Unmixing molecular agents from absorbing tissue in multispectral optoacoustic tomography. IEEE Trans MedImaging 2014; 33: 48–60. 10.1109/TMI.2013.2279994 24001986

[bib47] Cox B, Laufer JG, Arridge SR, Beard PC. Quantitative spectroscopic photoacoustic imaging: a review. J Biomed Opt 2012; 17: 061202. 22734732 10.1117/1.JBO.17.6.061202

[bib48] Jacques SL. Optical properties of biological tissues: a review. Phys Med Biol 2013; 58: R37–R61. 23666068 10.1088/0031-9155/58/11/R37

[bib49] Deán-Ben XL, Deliolanis NC, Ntziachristos V, Razansky D. Fast unmixing of multispectral optoacoustic data with vertex component analysis. Opt Laser Eng 2014; 58: 119–125.

[bib50] Kramer CM, Sinusas AJ, Sosnovik DE, French BA, Bengel FM. Multimodality imaging of myocardial injury and remodeling. J Nucl Med 2010; 51: 107s–121s. 20395347 10.2967/jnumed.109.068221 PMC3078824

[bib51] Nie LM, Chen XY. Structural and functional photoacoustic molecular tomography aided by emerging contrast agents. Chem Soc Rev 2014; 43: 7132–7170. 24967718 10.1039/c4cs00086b PMC4569000

