# Supplementary material for: Spiral volumetric optoacoustic tomography visualizes multi-scale dynamics in mice
Source: Light Sci Appl. 2017 Apr 7;6(4):e16247–. doi: 10.1038/lsa.2016.247 (PMC6062167; doi:10.1038/lsa.2016.247)
Supplement: Supplementary File [file lsa2016247x1.docx]

**SUPPLEMENTARY FIGURES**

**
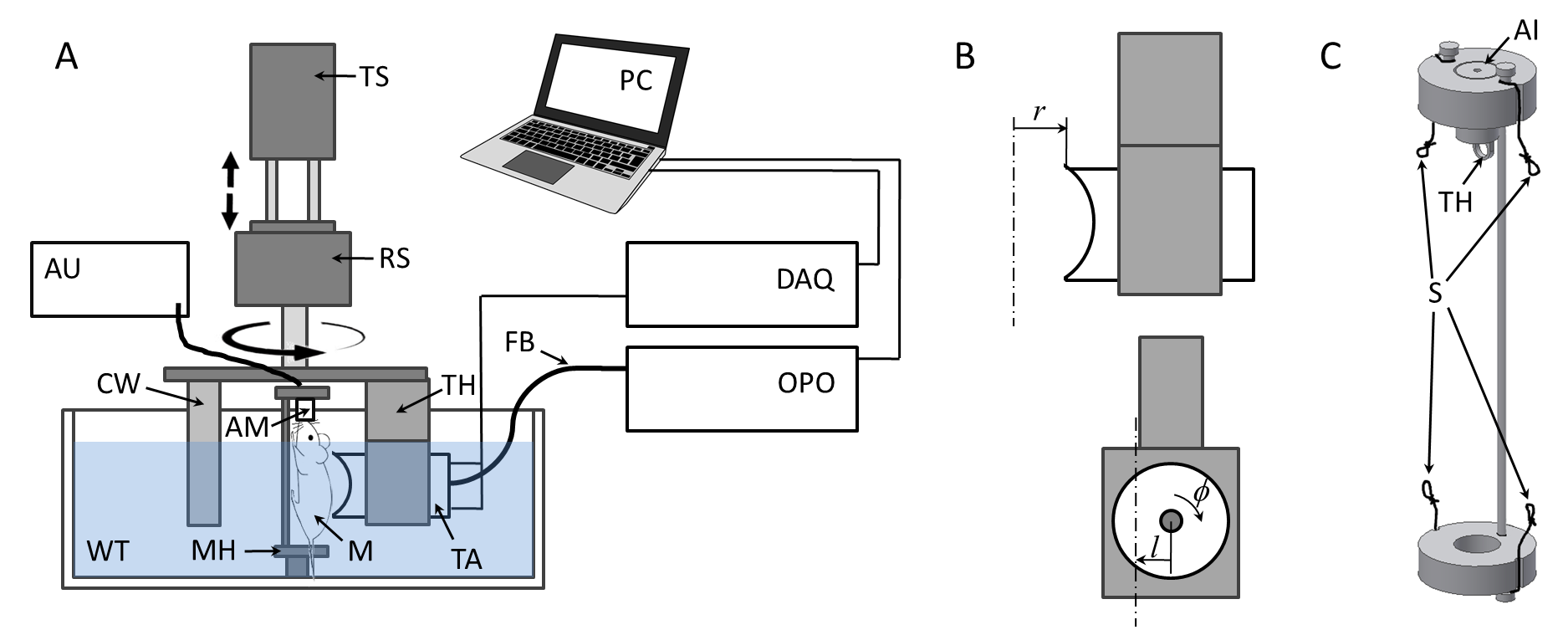
**

**Supplementary Figure 1**: (A) Lay-out of the SVOT setup. OPO - Optical parametric oscillator (laser), DAQ – Data acquisition, PC – Personal computer, TS – Translation stage, RS – Rotation stage, FB – Fiber bundle, TA – Transducer array, TH – Transducer holder, CW – Counter weight, AU – Anesthesia unit, AM – Anesthesia mask, WT – Water tank, M – Mouse, MH – Mouse holder. (B) Parameters used to calibrate the position of the transducer array relative to the rotation axis. (C) Lay-out of the mouse holder. AI – Anesthesia inflow, S – Strings, TH – teeth holder.

**
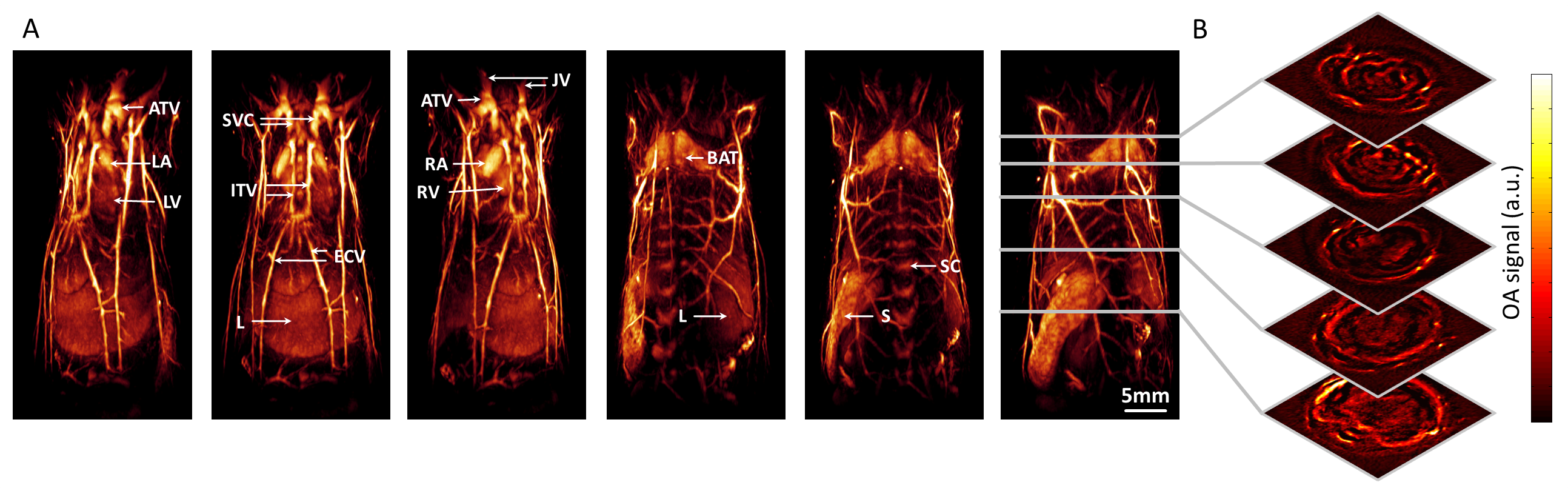
**

**Supplementary Figure 2**: (A) Different projection views of the 3D whole-body optoacoustic anatomical images acquired at 800 nm illumination wavelength. L – Liver, S – Spleen, SC – Spinal cord, BAT – Brown adipose tissue, LA – Left atrium, RA – Right atrium, LV – Left ventricle, RV – Right ventricle, EJV – External jugular veins, ATV – Auxiliary thoracic veins, SVC – Superior vena cava, ITV – Internal thoracic veins, ECV – Epigastrica cranialis veins. (B) Cross-sectional slices marked in (A).

**
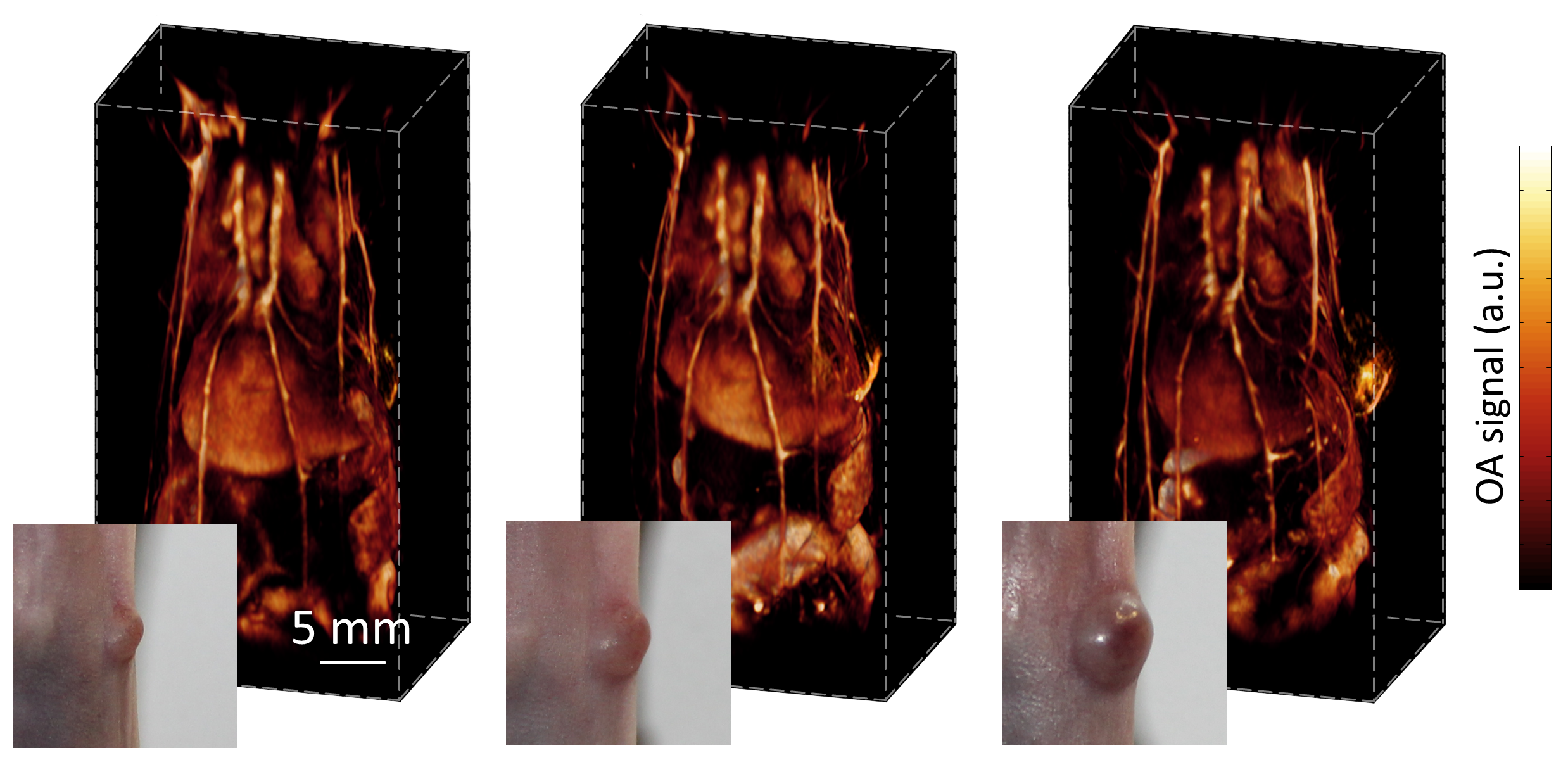
**

**Supplementary Figure 3**: 3D views of the whole-body optoacoustic anatomical images for 800 nm illumination wavelength acquired 6, 8 and 11 days after subcutaneous inoculation of 0.5·10^6^ 4T1 murine breast cancer cells into the thoracic mammary fat pad. Actual photographs of the tumor taken after the imaging experiments are also shown.

**
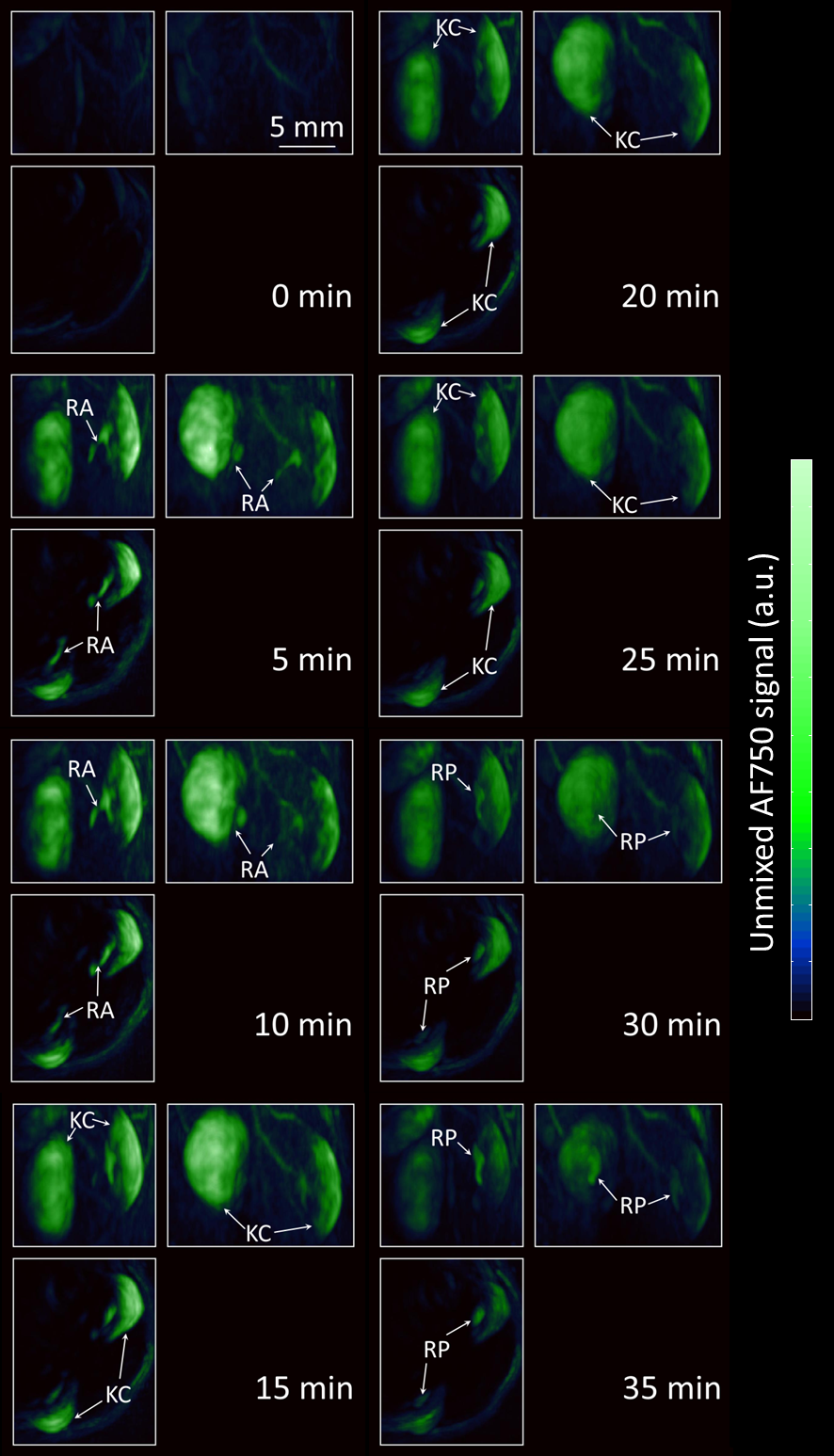
**

**Supplementary Figure 4**: Maximum intensity projections (MIPs) along *x*, *y* and *z* of the multi-spectrally-unmixed distribution of AF750 after tail-vein injection of 10 nmol at *t*=0 sec. RA – Renal artery, KC – Kidney cortex, RP – Renal pelvis.

**SUPPLEMENTARY MOVIES**

**Supplementary Movie 1.** Rotating (front) view of the 3D whole-body optoacoustic image acquired from a living mouse at 800 nm illumination wavelength.

**Supplementary Movie 2.** Rotating (rear) view of the 3D whole-body optoacoustic image acquired from a living mouse at 800 nm illumination wavelength.

**Supplementary Movie 3.** Axial, sagittal and coronal sections of the 3D whole-body optoacoustic image acquired from a living mouse at 800 nm illumination wavelength.

**Supplementary Movie 4.** Rotating view of the 3D image of a beating mouse heart superimposed onto the whole-body reference image. All data was acquired at 800 nm illumination wavelength. The movie was slowed down to 25 frames per second for better visualization, but the actual volumetric data were acquired at the full 100 Hz rate.

**Supplementary Movie 5.** Rotating view of the 3D distribution of ICG in the tumor area (green) superimposed onto the whole-body optoacoustic anatomical reference. Tail-vein injection of 100 nmol of the agent was done right at the starting point of the movie whose entire duration corresponds to 110 sec.
